# Integrated multi-omics reveals metabolic determinants of CRAB ST2 airway infection progression

**DOI:** 10.1128/spectrum.00195-25

**Published:** 2025-04-16

**Authors:** DingYun Feng, JianXia Zhou, Logen Liu, Ying Li, RongHua Zhong, WenBin Wu, WenZheng Zheng, TianTuo Zhang

**Affiliations:** 1 Department of Pulmonary and Critical Care Medicine, Institute of Respiratory Diseases of Sun Yat-Sen University, Third Affiliated Hospital of Sun Yat-Sen University 144991 , Guangzhou, China; 2 Clinical Research Center, The Second Affiliated Hospital, Hengyang Medical School, University of South China 159373, Hengyang, China; 3 National Health Commission Science and Technology Innovation Platform for Nutrition and Safety of Microbial Food, Guangdong Provincial Key Laboratory of Microbial Safety and Health, State Key Laboratory of Applied Microbiology Southern China, Guangdong Academy of Sciences, Institute of Microbiology 230784, Guangzhou, China; Institut National de Santé Publique du Québec, Sainte-Anne-de-Bellevue, Québec, Canada

**Keywords:** metabolomics, *Acinetobacter baumannii*, colonization, infection

## Abstract

*Acinetobacter baumannii* commonly causes lower airway colonization and infection and is easily confused. This study aimed to analyze the biological characteristics of carbapenem-resistant *A. baumannii* (CRAB) ST2 in the lower airway and identify an effective method for distinguishing between *A. baumannii* colonization and infection. Lower airway CRAB ST2 isolated at the Department of Respiratory and Critical Care Medicine and intensive care unit of our hospital from January 2021 to June 2023 were included, and their whole genome, biofilm-forming ability, bacterial virulence, and metabolome were analyzed. Fifty-six strains of CRAB with ST2 were identified, of which 32 were infection strains and 24 were colonization strains. The virulence and resistance genes, as well as the virulence and biofilm-forming ability, of ST2-type carbapenem-resistant lower airway infecting and colonizing *A. baumannii* strains were similar. The levels of metabolites were significantly lower in ST2-type carbapenem-resistant lower airway-infecting *A. baumannii* infection strains than those in the lower airway-colonizing strains. The levels of (S)-(+)−2-(aniline methyl) pyrrolidine, valine, ketoleucine, L-isoleucine, homoserine, *N*-acetyl-L-aspartate, and 2-aminoethanol-1-phosphate in the lower airway infection strains were significantly lower than those in the lower airway colonization strains. Bacterial virulence tests and biofilm formation ability could not distinguish the same ST of CRAB in the lower airway from the colonization or infection strains; however, metabolomics could. The biosynthesis and degradation pathways of valine, leucine, and isoleucine were downregulated, and changes in their metabolism may be important factors in promoting carbapenem-resistant *A. baumanniiCRAB* transformation from colonization to infection.

**IMPORTANCE:**

Carbapenem-resistant *A. baumannii* (CRAB) poses a critical threat in clinical settings, particularly due to challenges in distinguishing airway colonization from active infection, which complicates treatment decisions. This study highlights the limitations of conventional approaches—such as virulence gene profiling, phenotypic virulence assays, and biofilm formation analysis—in differentiating CRAB ST2 strains isolated from lower airway infections versus colonization. By integrating metabolomics, we identified distinct metabolic signatures linked to infection, including significant downregulation of valine, leucine, and isoleucine biosynthesis/degradation pathways and reduced levels of key metabolites (e.g., ketoleucine and L-isoleucine) in infection strains. These findings provide the first evidence that metabolic dysregulation may drive CRAB’s transition from colonization to invasive disease. This work advances our understanding of CRAB pathogenicity and offers a novel, metabolism-based strategy to improve diagnostic accuracy, guide targeted therapies, and optimize antimicrobial stewardship in managing CRAB-associated respiratory infections.

## INTRODUCTION

*Acinetobacter baumannii* is one of the most common pathogens that cause hospital-acquired infections, including pneumonia, urinary tract infections, and bloodstream infections (1, 2). Lim et al. found that the mortality rate of pneumonia caused by *A. baumannii* infection was as high as 42.6%, confirming that the severe challenge of pneumonia caused by *A. baumannii* infection significantly affects patient survival (3). In their prospective cohort study, John et al. included 129 patients with hospital-acquired infections, including 73.6% with ventilator-associated pneumonia and 60.4% with *A. baumannii* infection. The mortality rate associated with *A. baumannii* infection was 57.6%. This further supports the high morbidity and mortality rates associated with *A. baumannii* infections in critically ill patients (4).

The persistence of *A. baumannii* in the clinical environment enables it to acquire pathogenicity through constant contact with potential hosts, thereby repeatedly colonizing or infecting the host. Currently, the pathogenic mechanism of *A. baumannii* is believed to be mainly related to its biological characteristics, including adhesion, motility, and biofilm-forming ability (5). Notably, *A. baumannii* has a strong adhesion ability, can easily colonize the surface of the host or object, and is difficult to remove. Simultaneously, it can easily change from the colonization state to the infection state (6). Currently, the rapid identification of clinically isolated *A. baumannii* is lacking. Understanding the underlying mechanism by which *A. baumannii* acquires pathogenicity and transforms into infective bacteria is lacking, resulting in increased difficulty in preventing and controlling its infection. Bartal et al. found that carbapenem-resistant *A. baumannii* (CRAB) easily colonized both nonsterile (skin) and relatively sterile (respiratory tract) environments. If the host has obvious symptoms of infection (fever, cough, and sputum), physical signs (wet rales or rales), or laboratory results (such as elevated white blood cells, inflammatory markers, or imaging abnormalities), it is considered an *A. baumannii* respiratory infection (7).

Amino acids are key nutrients in the proliferation of bacteria and are also crucial substances that assist bacteria in evading host immune defenses. After bacteria infect a host, not only do their own amino acid metabolisms change, but they may also cause changes in the host’s amino acid metabolism (8). Some studies have found that energy metabolism disorders and the lack of energy substances, such as adenosine triphosphate (ATP), may be important causes of carbapenem resistance in *A. baumannii* (9). BCAA degradation pathways, regulated by the *abaI/abaR* QS system, play a role in *A. baumannii* virulence by influencing membrane structure, energy metabolism, and host interaction mechanisms (10). Some studies have found that under conditions of extreme amino acid starvation, the natural transformation mechanism of *Micrococcus luteus* can be shut down, which may be a response to amino acid starvation (11). However, there are currently no studies differentiating *A. baumannii* infection or colonization based on amino acid metabolism.

Therefore, this study aimed to analyze the biological characteristics of CRAB ST2 in the lower airway and identify an effective method for distinguishing between *A. baumannii* colonization and infection.

## MATERIALS AND METHODS

The strains were obtained from the culture of patients’ lower airway specimens (tracheal aspirates or bronchoalveolar lavage fluids). The related inpatients aged ≥18 years in the Department of Pulmonary and Critical Care Medicine, intensive care unit, and surgical intensive care unit of our hospital were included in our study from September 2021 to June 2023. This study was approved by the Ethics Review Committee of our Institute (approval no. RG2023-178-01) and was performed in strict accordance with the principles of the Declaration of Helsinki.

Bacterial sample preparation, gas chromatography-mass spectrometry (GC-MS) analysis, and *Galleria mellonella* virulence assay were performed using protocols used in our previous study (12). Whole-genome sequencing and biofilm-forming ability were assessed as previously described (13, 14). Strain typing MLST software was used to analyze all *A. baumannii* strains according to Pasteur typing standards.

Notably, *A. baumannii* infection strains were defined as those isolated from the patient’s lower airway and simultaneously confirmed by a hospital-acquired pneumonia diagnosis. The criterion for a diagnosis of hospital-acquired pneumonia is a new pulmonary infiltrate (occurring ≥48 hours after admission) associated with at least one of the following: new or increased cough with or without purulent tracheobronchial secretion or new pathogenic bacteria isolated from sputum or tracheal aspirate culture with ≥10^4^ colony-forming units per mL, fever (>37.8°C), leukocytosis, left shift, or leukopenia based on local normal values (14). Strains that did not meet the infection strain criteria were defined as colonization strains. All *A. baumannii* strains were confirmed as solely *A. baumannii* through culture, biochemical identification, and whole-genome sequencing.

Bacterial virulence assay: *Galleria mellonella* larvae of similar length, weight, and developmental stage were selected. The larvae were injected intra-abdominally with *A. baumannii* bacterial suspensions, incubated in a constant-temperature chamber, and observed for mortality over 96 hours. Strains demonstrating ≥80% lethality were classified as high-virulence strains, while those with <80% lethality were defined as low-virulence strains.

Biofilm formation capacity (15): Mature *A. baumannii* biofilms were cultured, and the optical density (OD) of each well was measured at 570 nm using a microplate reader. Biofilm maturity was assessed based on the absorbance values. The reference threshold (*A*_*0*_) was defined as the mean OD value (0.066) of nine negative control samples. Evaluation criteria: no biofilm formation (*A*_*x*_ ≤*A*_*0*_), weak positive (*A*_*0*_＜*A*_*x*_ ≤2 *A*_*0*_), positive (2 *A*_*0*_＜*A*_*x*_ ≤4 *A*_*0*_), and strong positive (4 *A*_*0*_＜*A*_*x*_).

This study utilized GC-MS, with the main procedures comprising metabolite extraction from bacterial samples, derivatization of metabolites, data acquisition, preprocessing of GC-MS data, screening of differential metabolites, and pathway enrichment analysis.

### Software used for bioinformatics analysis of *A. baumannii*

The bioinformatics analysis processes involved in this study include splitting and quality control of sequencing data, whole-genome assembly of *A. baumannii*, quality control and annotation, analysis of virulence and drug resistance of strains in different groups, genomic evolutionary analysis of *A. baumannii* in different groups, and comparative genomic analysis of strains in different groups. The software used included Bcl2fastq, Trimmomatic, FastQC, SPAdes, QUAST, Prokka, Abricate, MLST, and Parsnp.

### Statistical analysis

For the metabolomic data processing, normalization and analysis were carried out with the assistance of multiple software tools, including IBM SPSS Statistics 22, Simca-P+12.0, GraphPad Prism 8.3, and R software (version R4.2.3). The enrichment analysis of pathways was executed using MetaboAnalyst 5.0, which can be accessed at https://www.metaboanalyst.ca. GraphPad Prism 8.3 was employed to analyze the experimental data, and the results were presented in the form of mean ± standard deviation. To compare the differences either among multiple groups or between two groups, one-way analysis of variance or the *t*-test was utilized. Adobe Illustrator CC 2018 was used to integrate figures. Regarding the survival rates of *G. mellonella*, they were depicted through survival curves. Each experiment was replicated a minimum of three times. Statistical significance was determined when the *P*-value was less than 0.05.

## RESULTS

In total, 67 *A*. *baumannii* strains were isolated from the lower airways of patients. Among these, 56 strains were CRAB with ST2, of which 32 were infection strains and 24 were colonization strains.

The virulence genes *abaR*, *bauA*, *clpV/tssH*, *hcp/tssD*, *tagX*, *tssA*, *tssB*, *tssC*, *tssE*, *tssF*, *tssG*, *tssK*, *tssL*, *algW*, and *tssM* were not significantly different between lower airway infection and colonization strains (Table 1).

**TABLE 1:** Comparison of virulence genes between lower airway CRAB infection and colonization strains

Virulence gene	Infection strains	Colonization strains	*P*
	*n* = 32	*n* = 24	
*abaR*	32 (100%)	24 (100%)	1
*algW*	0	0	1
*bauA*	31 (96.9%)	24 (100%)	1
*clpV/tssH*	31 (96.9%)	23 (95.8%)	1
*hcp/tssD*	31 (96.9%)	23 (95.8%)	1
*tagX*	31 (96.9%)	23 (95.8%)	1
*tssA*	31 (96.9%)	23 (95.8%)	1
*tssB*	31 (96.9%)	23 (95.8%)	1
*tssC*	31 (96.9%)	23 (95.8%)	1
*tssE*	31 (96.9%)	23 (95.8%)	1
*tssF*	31 (96.9%)	24 (100%)	1
*tssG*	32 (100%)	23 (95.8%)	0.429
*tssK*	31 (96.9%)	23 (95.8%)	1
*tssL*	31 (96.9%)	23 (95.8%)	1
*tssM*	31 (96.9%)	23 (95.8%)	1

There were no significant differences in the carrier status of the common drug resistance genes *OXA-66*, *ADC-73*, *APH(3 ")-Ib*, *APH(6)-Id*, *tet(B)*, *OXA-23*, *armA*, *msrE*, and *mphE* between carbapenem-resistant lower airway-infecting *A. baumannii* strains and the colonization strains (Table 2).

**TABLE 2:** Comparison of drug resistance genes between lower airway CRAB infection and colonization strains

Drug resistance gene	Infection strains	Colonization strains	*P*
	*n* = 32	*n* = 24	
*OXA-66*	30 (93.8%)	22 (91.7%)	1
*ADC-73*	27 (84.4%)	19 (79.2%)	0.730
*APH(3'')-Ib*	30 (93.8%)	24 (100%)	0.501
*APH(6)-Id*	30 (93.8%)	24 (100%)	0.501
*tet(B*)	30 (93.8%)	24 (100%)	0.501
*OXA-23*	30 (93.8%)	23 (95.8%)	1
*armA*	30 (93.8%)	23 (95.8%)	1
*msrE*	30 (93.8%)	23 (95.8%)	1
*mphE*	30 (93.8%)	23 (95.8%)	1

Of the 56 lower airway CRAB ST2 strains in this study, 21 (including 15 lower airway infection strains and 6 lower airway colonization strains) were randomly selected for the bacterial virulence test and the determination of biofilm formation ability. The results showed that there were six (40%) and three highly virulent strains (50%) among the lower airway infection and colonization strains, respectively, indicating no significant difference in mortality and virulence between the lower airway infection and colonization strains. There were five strains (33.3%) and one strain (16.7%) with a strong positive biofilm-forming ability among the lower airway infection and colonization strains, respectively. There was no significant difference in the biofilm-forming ability between the two groups (Table 3).

**TABLE 3:** Virulence and biofilm formation abilities of 21 lower airway CRAB strains

Content	Infection strains	Colonization strains	*P*
	*n* = 15	*n* = 6	
*Galleria mellonella* virulence assay			
Highly virulent strains	6 (40%)	3 (50%)	1
Low-virulence strains	9 (60%)	3 (50%)	1
Biofilm-forming ability			
Strong positive	5 (33.3%)	1 (16.7%)	0.623
Positive	1 (0.67%)	3 (50.0%)	0.053
Weak positive	9 (60.0%)	2 (33.3%)	0.361

**Fig 1:**
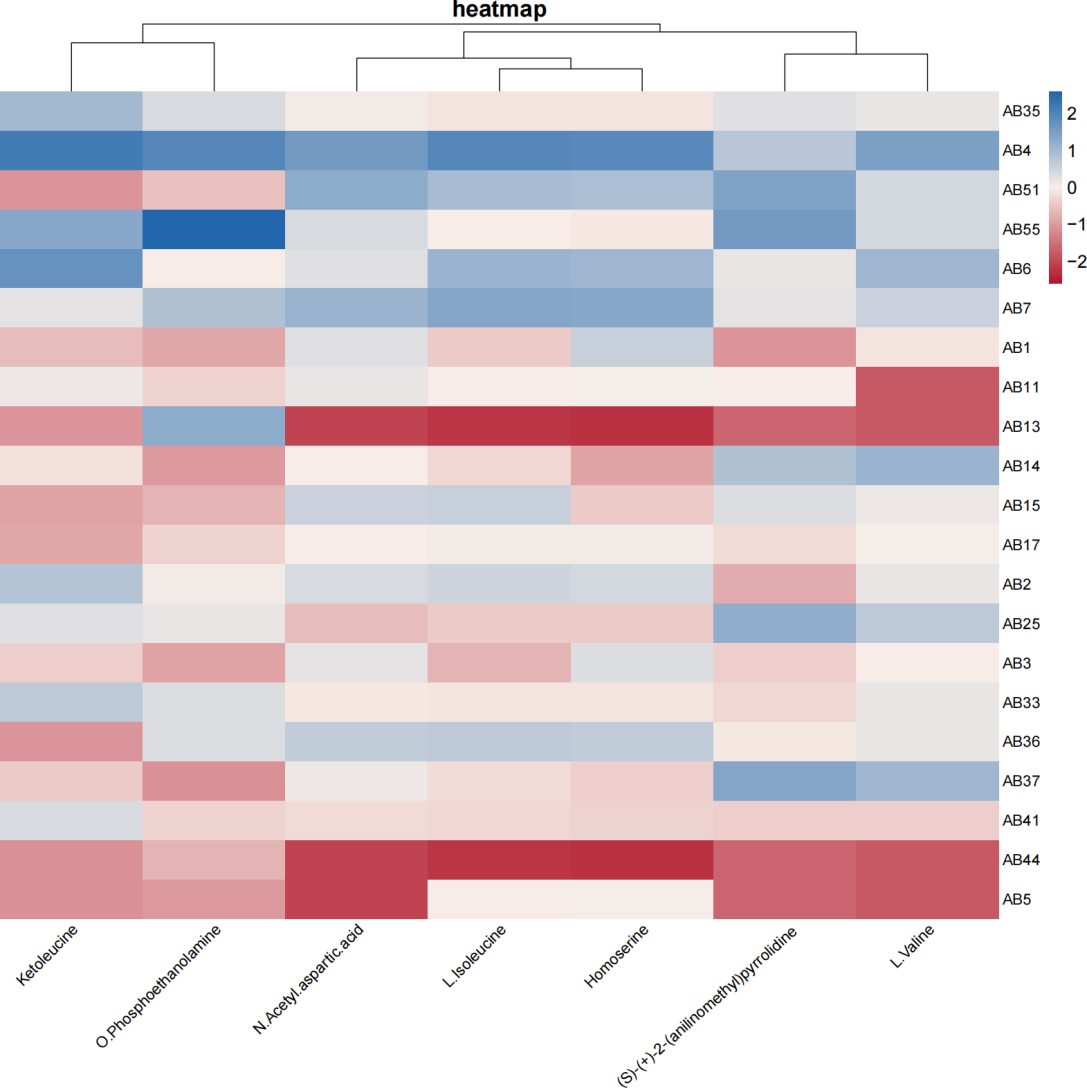
Comparison of cluster heat maps of metabolites of lower airway CRAB infection and colonization strains. Note: from top to bottom, AB35–AB7 are colonization strains (6), and AB1–AB5 are infection strains (15).

Notably, 1,067 metabolites from 21 strains were analyzed, and 107 different metabolites were found in the initial screening between the two groups. The final seven metabolites were statistically different (Table 4). The levels of (S)-(+)−2-(aniline-methyl) pyrrolidine, valine, ketoleucine, L-isoleucine, homoserine, *N*-acetyl-L-aspartate, and 2-aminoethanol-1-phosphate in the CRAB infection strains were significantly lower than those in the colonization strains (Fig. 1).

**TABLE 4:** Comparison of metabolome levels of lower airway CRAB strains and colonization strains

Metabolites	Infection strains	Colonization strains	*P*
	*N* = 15	*N* = 6	
(S)-(+)−2-(aniline-methyl) pyrrolidine	406,032.44 ± 290,888.41	715,251.07 ± 187,148.02	0.027
Valine	155,959.16 ± 105,159.77	256,308.08 ± 49,033.46	0.039
Ketoleucine	254,343.44 ± 231,762.71	682,750.90 ± 395,913.45	0.006
L-isoleucine	4,621,816.54 ± 2,111,937.84	7,588,565.00 ± 1,961,090.40	0.008
Homoserine	4,704,291.19 ± 2,180,638.11	7,575,892.00 ± 1,956,496.71	0.011
*N*-acetyl-L-aspartate	22,789,613.94 ± 12,344,379.07	37,180,635.00 ± 7,999,527.10	0.017
2-aminoethanol-1-phosphate	3,679,499.59 ± 3,078,418.16	9,227,148.00 ± 5,560,133.97	0.008

The screened differential metabolites were submitted to the Omics Bean website for metabolite pathway enrichment analysis (Kyoto Encyclopedia of Genes and Genomes, KEGG). The results showed that CRAB infection and colonization strains in the lower airway may differ in the following 10 metabolic pathways: biosynthesis and degradation of valine, leucine, and isoleucine; cyanamide metabolism; aminoacyl biosynthesis; ATP-binding cassette (ABC) transport proteins; pantothenic acid and coenzyme biosynthesis; sulfur metabolism; lysine biosynthesis; glycine, serine, and threonine metabolism; and glycerol phospholipid metabolism (Fig. S1).

The KEGG pathway map was analyzed to compare the enrichment of differential metabolites in the lower airway infection and colonization strains, and the differential metabolites were labeled in the KEGG pathway map. Downregulated differential metabolites were observed during the biosynthesis (Fig. S2) and degradation (Fig. S2) of valine, leucine, and isoleucine.

**Fig 2:**
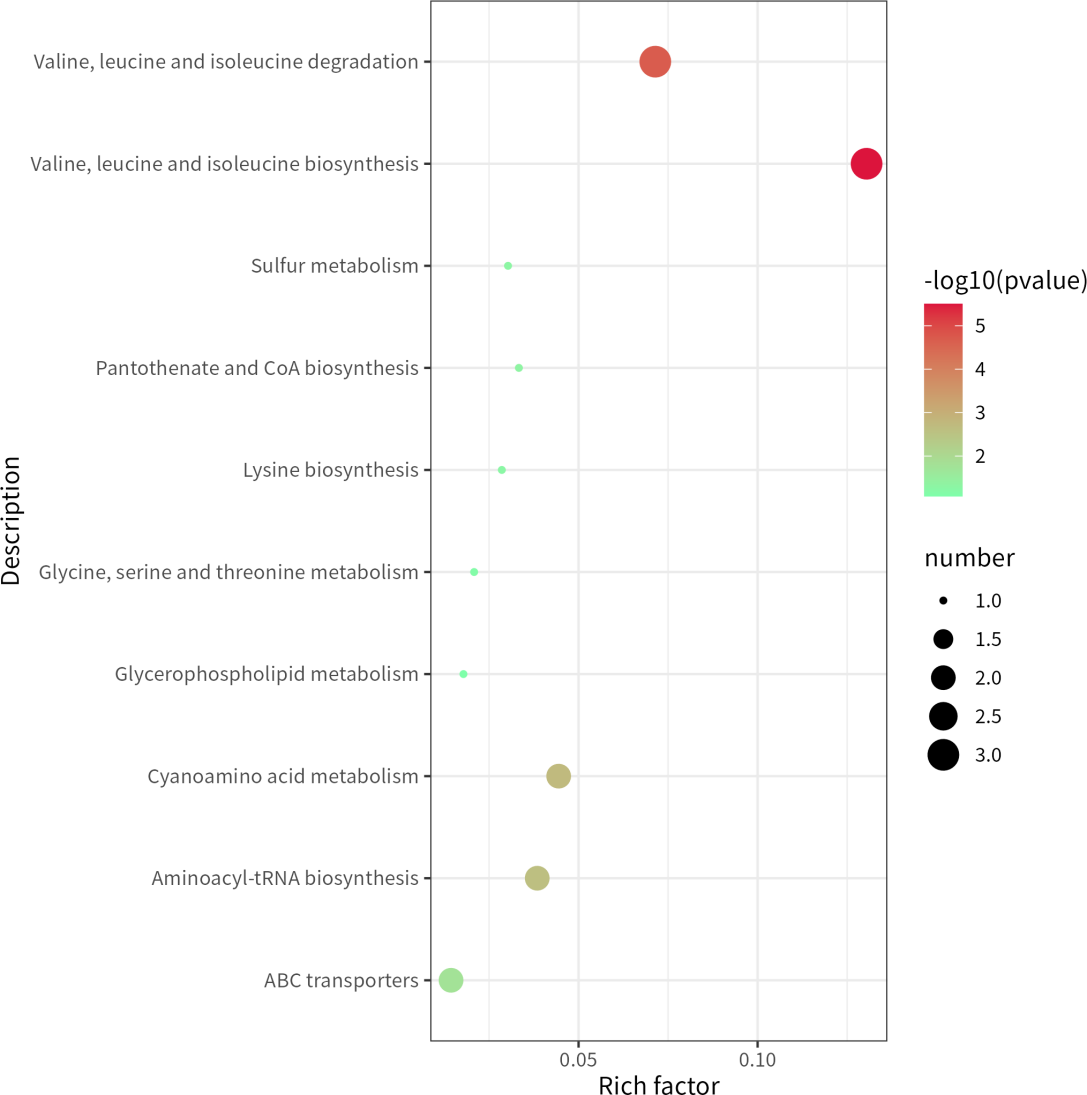
KEGG pathway enrichment (bubble diagram).

The bubble diagram of the KEGG enrichment pathway (Fig. 2) revealed that the degradation and biosynthesis pathway enrichment of valine, leucine, and isoleucine were the most significant, and the biosynthesis pathway enrichment of valine, leucine, and isoleucine was the strongest.

## DISCUSSION

The repeated occurrence of CRAB infections in the same hospital ward is common. A 2024 study conducted in Greek tertiary hospitals found, through whole-genome sequencing and pulsed-field gel electrophoresis, that ventilators and ventilator stands are the main environmental reservoirs of carbapenemase-resistant *A. baumannii* (positive rate of 19.9%). Cross-contamination between patients and the environment within the same ward leads to the persistence of resistant strains, which are difficult to completely eradicate even with disinfection measures (16). In studying an *OXA-23* carbapenemase-resistant *A. baumannii* infection outbreak in a skilled care facility, Smith et al. confirmed the importance of whole-genome sequencing and the idea that shared equipment can spread strains (17). In clinical practice, *A. baumannii* strains with the same drug-resistant phenotype often show different states in different hosts or even within the same host, manifesting as either infection or colonization strains. Previous studies have suggested that the virulence gene-carrying, CRAB strains resulting in ventilator-associated pneumonia are more abundant than those of CRAB strains colonized in the lower airway; however, the ST types of the strains included in this study are diverse, which is somewhat different from the ST2 CRAB strains selected in this study (18). In this study, there were no statistically significant differences in the *abaR*, *algW* (related to adhesion), *hcp/tssD* (related to the secretion system), *TagX*, or *TssABCEGKLM* gene affecting the surface motility of the two groups of strains. There were no significant differences in drug resistance genes, including *OXA-66*, *OXA-23*, *msrE*, *mphE*, *tet(B*), *rmA*, *APH(6)-Id*, and *APH(3 ")-Ib*. This indicated that in the CRAB strains isolated from the lower airway, there were no significant changes in the virulence and drug resistance genes of the infection and colonization strains, which was similar to previous studies (19).

In this study, there was no significant difference in the virulence and biofilm-forming ability between the infection and colonization strains of ST2 CRAB isolates from the lower airway. Studies have focused on how *A. baumannii* maintains its virulence for a long time and found that the virulence of *A. baumannii* infection strains is similar to that of the colonization strains identified on the surface of hospital equipment, with no significant differences between these strains (20). Other studies have found that *A. baumannii* infection strains isolated from wounds of patients or ischemic wounds are more virulent than the *A. baumannii* colonization strains isolated from patient wounds (21). The biofilm-forming ability of *A. baumannii* is generally associated with drug resistance (22, 23). In this study, comparison of the differences between CRAB infection and colonization strains isolated from the lower airway showed that the drug-resistant phenotypes of the included strains were consistent. Therefore, the biofilm-forming abilities of the lower airway *A. baumannii* infection and colonization strains were similar, which was consistent with the common biological characteristics of the strains.

This study confirmed that there are significant differences between the metabolome of CRAB infection and colonization strains isolated from the lower airway. No similar studies have been reported thus far. Studies have indicated that kojic acid metabolism may be associated with the colonization of *A. baumannii* by influencing biofilm formation, including quorum sensing, membrane assembly, bacterial virulence, and metabolic plasticity (24), indicating that metabolic changes can also reflect the colonization status of *A. baumannii*. As branched-chain amino acids, valine and isoleucine are important nutrients for bacteria and are involved in protein synthesis, signal transduction, and the fine-tuning of adaptation to amino acid hunger. In some pathogenic bacteria, adaptation to amino acid starvation involves inducing virulence gene expression, and branched-chain amino acids not only support proliferation during bacterial infection but also support host defense evasion (25). The natural transformation mechanism of *Micrococcus luteus* has been shown to shut down when it is in a state of extreme amino acid hunger, which may be a response to this stress (11). In this study, according to the KEGG pathway map analysis, the metabolites of the biosynthesis pathways of valine, leucine, and isoleucine and the degradation pathways of valine, leucine, and isoleucine were downregulated in the above differential metabolic pathways. This suggests that the lower airway infection strains may have decreased the biosynthesis and degradation of valine, leucine, and isoleucine. The concentrations of valine, leucine, and isoleucine biosynthesis pathways were the strongest in combination with the bubble map analysis of the KEGG enrichment pathway; that is, the reduction of valine, leucine, and isoleucine biosynthesis in lower airway infection strains was significantly greater than the reduction in degradation. Therefore, lower airway CRAB infection strains may have both reduced intake and consumption during the pathogenic period and maintain their virulence by reducing consumption.

### Conclusion

Assessment of bacterial virulence and biofilm formation ability could not distinguish between the colonization and infection states of ST2 CRAB in the lower airway; however, metabolomics can distinguish between multidrug-resistant *A. baumannii* colonization and infection. The biosynthesis and degradation pathways of valine, leucine, and isoleucine were downregulated, and metabolic changes may play important roles in their transformation from colonization to infection.

## Data Availability

The whole-genome sequencing and metabolomic raw data were deposited to Zenodo under identifier https://doi.org/10.5281/zenodo.15170990

## SUPPLEMENTAL MATERIAL

Fig. S1 spectrum.00195-25-s0001.pdf KEGG analysis of the metabolites of carbapenem-resistant *A. baumannii* infection and colonization strains in the lower airway.

Fig. S2 spectrum.00195-25-s0002.pdf Pathways of valine, leucine, and isoleucine biosynthesis.

Fig. S3 spectrum.00195-25-s0003.pdf Valine, leucine, and isoleucine degradation pathways.

ASM does not own the copyrights to Supplemental Material that may be linked to, or accessed through, an article. The authors have granted ASM a non-exclusive, world-wide license to publish the Supplemental Material files. Please contact the corresponding author directly for reuse.
