# Supplementary material for: Integrated multi-omics reveals metabolic determinants of CRAB ST2 airway infection progression
Source: Microbiol Spectr. 2025 Apr 16;13(6):e00195-25. doi: 10.1128/spectrum.00195-25 (PMC12131721; doi:10.1128/spectrum.00195-25)
Supplement: Fig. S1 — KEGG analysis of the metabolites of carbapenem-resistant A. baumannii infection and colonization strains in the lower airway. [file spectrum.00195-25-s0001.pdf]

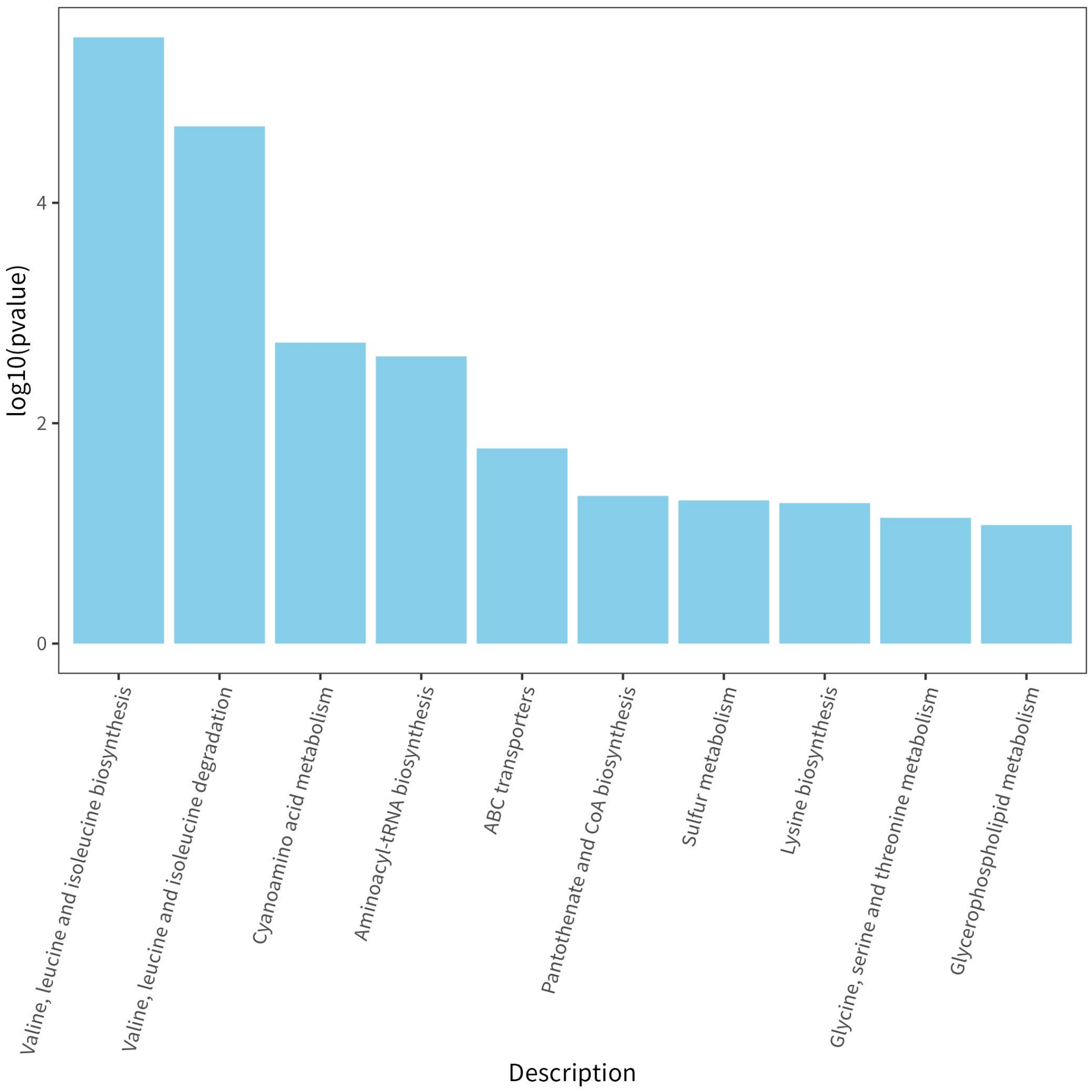

Figure S1. KEGG analysis of the metabolites of carbapenem-resistant *A. baumannii* infection and colonisation strains in the lower airway.
