# Supplementary figures and images for: Integrated multi-omics reveals metabolic determinants of CRAB ST2 airway infection progression
Source: Microbiol Spectr. 2025 Apr 16;13(6):e00195-25. doi: 10.1128/spectrum.00195-25 (PMC12131721; doi:10.1128/spectrum.00195-25)

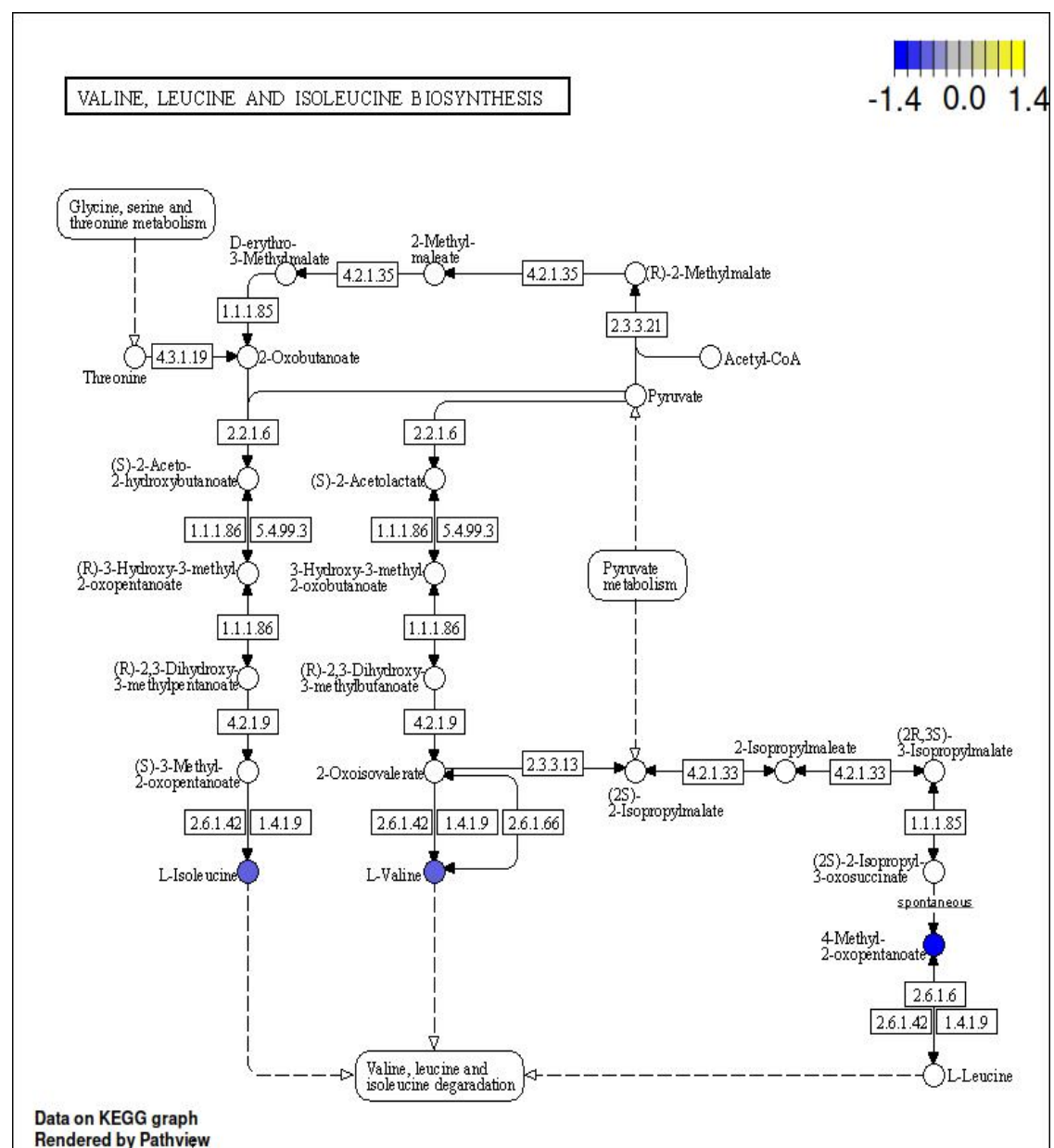

Figure S2 Pathways of valine, leucine, and isoleucine biosynthesis.

Supplement: Fig. S2 — Pathways of valine, leucine, and isoleucine biosynthesis. [file spectrum.00195-25-s0002.pdf]

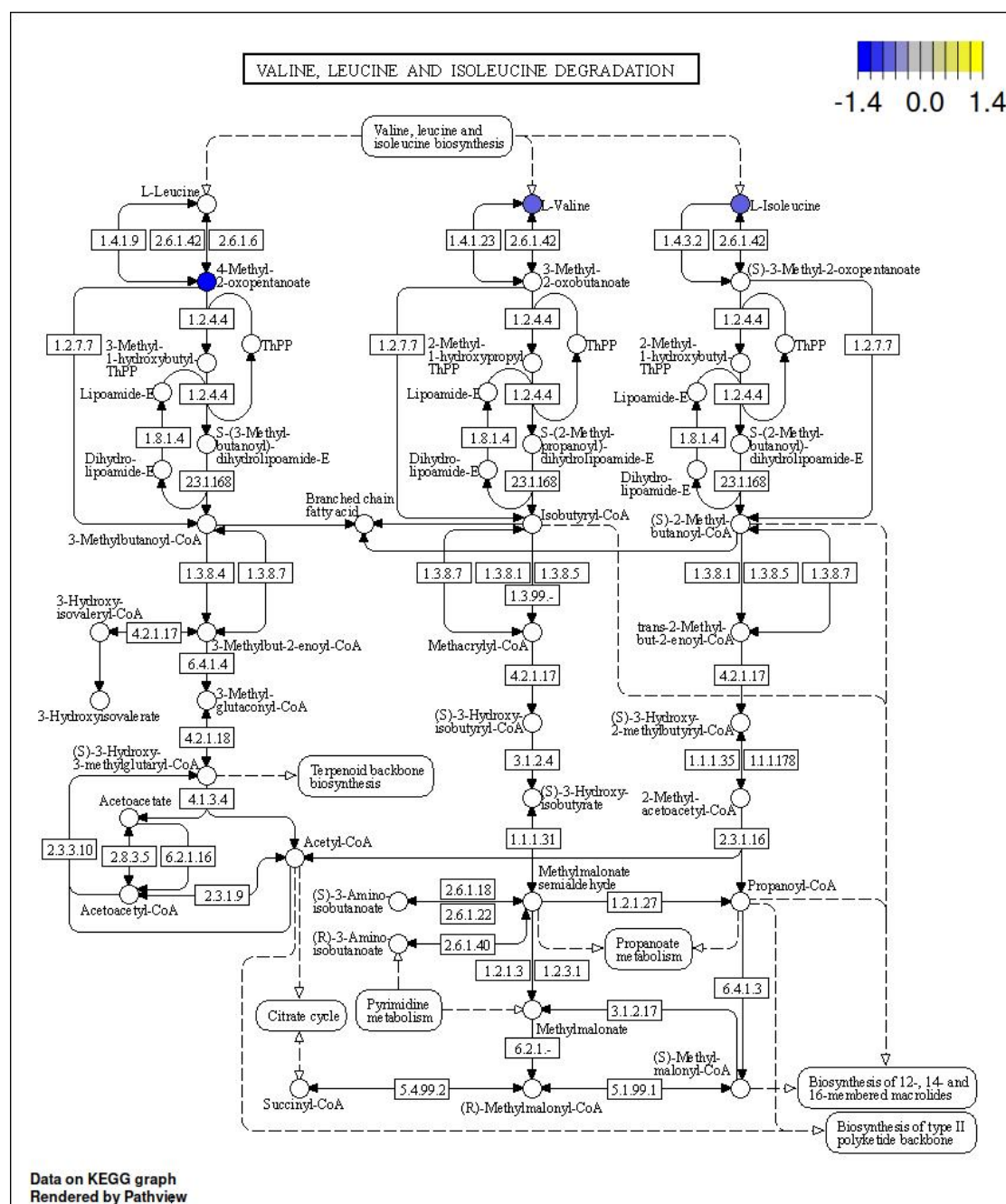

Figure S3. Valine, leucine, and isoleucine degradation pathways.

Supplement: Fig. S3 — Valine, leucine, and isoleucine degradation pathways. [file spectrum.00195-25-s0003.pdf]
